# Plasmonic ternary hybrid photocatalyst based on polymeric g-C_3_N_4_ towards visible light hydrogen generation

**DOI:** 10.1038/s41598-020-57493-x

**Published:** 2020-01-20

**Authors:** Yuping Che, Qingqing Liu, Bingxin Lu, Jin Zhai, Kefeng Wang, Zhaoyue Liu

**Affiliations:** 1 Key Laboratory of Bio-Inspired Smart Interfacial Science, Technology of Ministry of Education and Beijing Advanced Innovation Center for Biomedical Engineering, Beijing Key Laboratory of Bio-inspired Energy Materials and Devices, School of Chemistry, Beihang University, Beijing, 100191 P.R. China; 2 Henan Engineering Center of New Energy Battery Materials, College of Chemistry and Chemical Engineering, Shangqiu Normal University, Shangqiu, 476000 Henan P.R. China

**Keywords:** Photocatalysis, Hydrogen fuel

## Abstract

Surface plasmon resonance (SPR) effect of noble metal nanoparticles (NPs) for photocatalysis has a significant enhancement. In this system, a plasmonic ternary hybrid photocatalyst of Ag/AgBr/g-C_3_N_4_ was synthetized and used in water splitting to generation H_2_ under visible light irradiation. 18%Ag/AgBr/g-C_3_N_4_ showed the highest photoactivity, with the efficiency of hydrogen generation as high as 27-fold to that of pristine g-C_3_N_4_. Compared to simple mixture of Ag/AgBr and g-C_3_N_4_, hetero-composite Ag/AgBr/g-C_3_N_4_ showed a higher photoactivity, even though they contained same content of Ag/AgBr. We find that significant factors for enhancing properties were the synergistic effect between Ag/AgBr and g-C_3_N_4_, and the light absorption enhancing by SPR effect of Ag NPs. Ag/AgBr NPs firmly anchored on the surface of g-C_3_N_4_ and their high dispersion were also responsible for the improved activity and long-term recycling ability. The structure of Ag/AgBr/g-C_3_N_4_ hybrid materials and their enhancement to photocatalytic activity were discussed. Meanwhile, the possible reaction mechanism of this system was proposed.

## Introduction

Increasing attentions to environment and energy crises have spurred intense research on solar energy transform and utilization^1–3^. Hydrogen as an environmentally friendly energy source has gained more and more attention. Photocatalytic water splitting to produce hydrogen by harnessing sunlight holds particular promise as this process is economic and environmentally friendly^4–7^. Recent years, graphene-like carbon nitride (g-C_3_N_4_) material as a polymeric compound photocatalyst has attracted considerable attention for hydrogen generation and degradation ability of organic pollutants under visible light irradiation^8–13^. As a robust and stable visible-light-driven photocatalyst with the appropriate band energy, nontoxicity and abundance properties, it also has good chemical stability, attractive electronic structure and medium-bandgap of approximately 2.7 eV^8,14,15^. Meanwhile, g-C_3_N_4_ is easily-obtained via a one-step method from cheap feed stocks, such as cyanamide^16^, dicyandiamide^17^, melamine^18^, thiourea^19^ and urea^20^. Although g-C_3_N_4_ emerges as a good candidate for solar catalysis because of its unique physicochemical properties. While, g-C_3_N_4_ also suffers from some drawbacks, such as low visible-light utilization (only the light with the wavelength < 460 nm can be absorbed) and rapid recombination of photogenerated charges. Hence, various approaches have been proposed to overcome these glitches^21–25^, such as metallic and non-metallic doping, design of heterojunctions, morphology control, and construction of C or N defects. Among these, it is a key remedy to choose appropriate noble metals as co-catalyst^26,27^. Pt as a most efficient co-catalyst is used to modify g-C_3_N_4_ to enhance charge separation in photocatalytic process^28,29^. However, the scarcity and high cost of Pt seriously impede its extensive applications.

Recently, many studies have found that Ag as surface plasmon resonance (SPR) material which can be triggered by visible light is introduced into photocatalyst system. They can efficiently convert solar energy into chemical energy under visible light irradiation^30^. Meanwhile, the introduction of AgX (X = Cl, Br, I) to semiconductor photocatalysts can enhance photogeneration charge separation efficiency. Three-component plasmonic photocatalysts based on Ag/AgX (X = Cl, Br, I) have improved stability and photocatalytic performance of single photocatalyst^31–34^. For instance, photocatalysts decorated by Ag/AgCl exhibit excellent visible light absorption performance due to the synergistic effect of Ag/AgCl and SPR effect of Ag NPs^32,35^, Ag@AgCl^36^, Ag/AgCl/TiO_2_ nanotube arrays^37^ and Ag/AgCl/Al_2_O_3_^38^ showed high activity in degradation of organic pollutants (MO and MB) under visible light irradiation; Ag/AgBr hybrids display a synergistic effect between semiconductors and plasmonic metals and exhibit a considerably high photocatalytic performance for pentachlorophenol degradation^39^. The dispersion and stability of Ag/AgI can be further enhanced by compounding with other semiconductors^34^. Thus, developing semiconductor photocatalysts including inexpensive metals and their compounds with excellent light-trapping performance, high charge separation efficiency, and favorable recycling capabilities is extremely important^40–43^.

**Figure 1:**
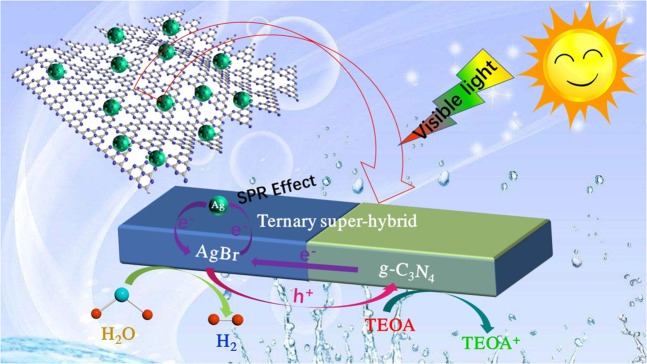
Schematic diagram of hydrogen production from Ag/AgBr/g-C_3_N_4_. (AgBr nanoparticles anchored by Ag nanoparticles are attached to g-C_3_N_4_ surface. Photogenerated electrons migrated to AgBr conduction band, and were enriched on the surfaces of AgBr nanoparticles to produce hydrogen on the surface of AgBr, while the holes were enriched in the g-C_3_N_4_ valence band, where they reacted with TEOA and OH^−^).

Considering the proper band gap and conduction band (CB) position of g-C_3_N_4_ and AgBr, we constructed a ternary photocatalyst with metal Ag and AgBr NPs supported on g-C_3_N_4_ (shown as Fig. 1). For the first time, we used this plasmonic ternary photocatalyst to generate H_2_ under visible light irradiation which demonstrated high efficiency for photocatalytic water splitting. In this system, room temperature ionic liquid ([Amin]Br) was used as Br^−^ source and space steric agent in preparing Ag/AgBr/g-C_3_N_4_ composite. Ag/AgBr NPs were highly dispersed on g-C_3_N_4_ nanosheets surface. This kind of distinctive Ag/AgBr/g-C_3_N_4_ ternary hybrid photocatalyst has substantially overcome the shortcomings compared with the single components and realized strong light absorption, high charge-separation efficiency, perfect photocatalytic stability, and relative strong redox ability^44,45^. The mechanism of Ag/AgBr/g-C_3_N_4_ hetero-composites photocatalysis was further discussed in details. Ag NPs with strong UV-vis absorption could be excited by visible incident light, thereby resulting in SPR effect in this heterostructure system to enhance light absorption. Considering band gap matching of AgBr and g-C_3_N_4_, the separation efficiency of photoexcited charges was considerably improved.

## Results and Discussion

### Morphology and component analysis

**Figure 2:**
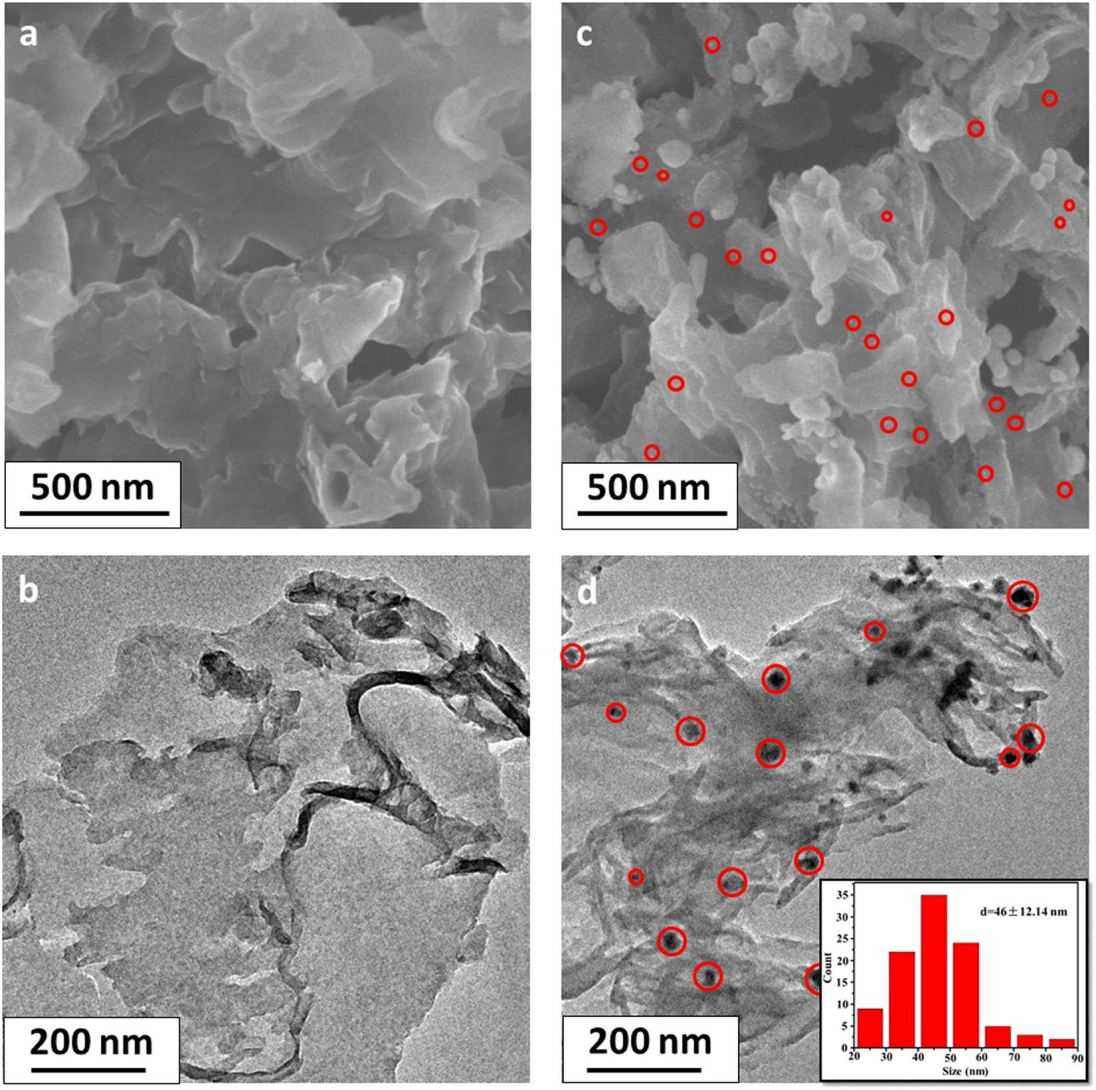
SEM images of (**a**) g-C_3_N_4_, (**c**) 18%Ag/AgBr/g-C_3_N_4_; TEM images of (**b**) g-C_3_N_4_, (**d**) 18%Ag/AgBr/g-C_3_N_4_ (Insert: the statistical size distribution of Ag/AgBr obtained from 100 nanoparticles).

The microscopic and structural morphologies of pure g-C_3_N_4_ and 18%Ag/AgBr/g-C_3_N_4_ were revealed by scanning electron microscope (SEM) and transmission electron microscopy (TEM), shown in Fig. 2. The representative structure of as-obtained g-C_3_N_4_ comprised large aggregates of 2-D nanosheet with irregular morphology and nonuniform dimensions (Fig. 2a). TEM image shown in Fig. 2b displayed smooth and flat layers in pure g-C_3_N_4_ sample. However, after ultrasonic treatment, some single nanosheets (Fig. 2c) emerged in g-C_3_N_4_ sample, although some aggregates were still present. After impregnation into AgNO_3_ and [Amim]Br solutions, the aggregated g-C_3_N_4_ was peeled into thin layers, and many AgBr NPs were inserted into the layers, as shown by red circles in Fig. 2c,d. This nanosheet microstructure with large specific surface area could provide more sites for formation of Ag/AgBr particles. SEM and TEM images of Ag/AgBr/g-C_3_N_4_ were obviously different from those of bare g-C_3_N_4_. Zero-dimensional Ag/AgBr NPs anchored on g-C_3_N_4_ surface formed heterojunction structure. As shown in Fig. 2d, Ag/AgBr NPs with the size ranging from 20 nm to 90 nm were dispersed well on the g-C_3_N_4_ surface with insignificant aggregation. At the same time, the particle size distribution was statistically analyzed. The statistical results showed that the size of Ag/AgBr particle was dispersed mainly in the range of 40 to 60 nm (shown as the insert in Fig. 2d). Such even dispersion indicated a strong anchoring effect of g-C_3_N_4_ to Ag/AgBr nanocrystals. Obtained Ag/AgBr/g-C_3_N_4_ nanostructure could fully utilize Ag/AgBr outer surfaces and the interfaces between Ag/AgBr and g-C_3_N_4_, which was very important to enhance photoactivity of the hybrid. However, when Ag/AgBr content reached 21%, the large amount of Ag/AgBr on g-C_3_N_4_ surface resulted in its agglomeration, as shown in Fig. S1d. The size of Ag/AgBr particles increased, which would decrease its photoactivity. Meanwhile, Ag/AgBr without g-C_3_N_4_ showed a large scale ranging from 100 nm to >700 nm with substantial agglomeration, as shown in Fig. S1e. And the photocatalysts with other contents were shown in Fig. S1a–c.

**Figure 3:**
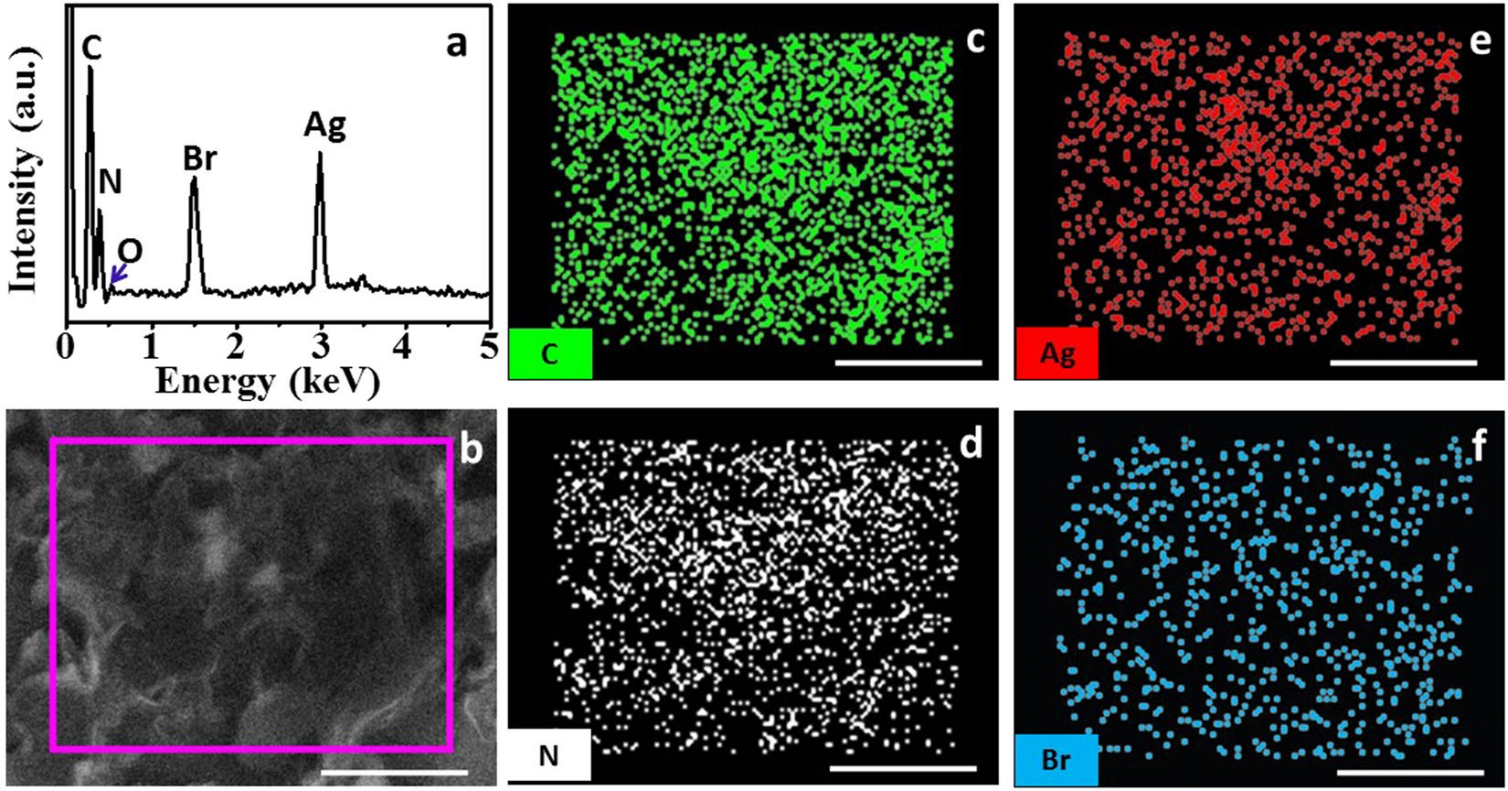
(**a**) EDS spectrum for 18%Ag/AgBr/g-C_3_N_4_; (**b**) SEM of 18%Ag/AgBr/g-C_3_N_4_, (**c**–**f**) EDS mapping for different elements of 18%Ag/AgBr/g-C_3_N_4_ nanocomposite (the scale bar is 500 nm).

Energy dispersive spectrometer (EDS) and elemental mapping data of 18%Ag/AgBr/g-C_3_N_4_ were also measured, shown in Fig. 3. The EDS result revealed that sample mainly contained four elements (i.e., C, N, Ag and Br) after removing other element introduced from environment. As shown in Fig. 3a, a small amount of O was detected due to the trace adsorption of O_2_ onto the sample surface. The weight percent of C, N, Ag, Br for 18%Ag/AgBr/g-C_3_N_4_ nanocomposite were theoretical calculated and quantitatively analyzed. Similar results were obtained (32.1, 49.9, 10.3 and 7.7%; 32.4, 49.3, 10.8, 7.1%, respectively). To verify the elemental distribution of obtained 18%Ag/AgBr/g-C_3_N_4_ hybrid, elemental mapping of the sample was also demonstrated, as shown in Fig. 3c–f. The results showed that Ag and Br were homogeneously distributed on g-C_3_N_4_ nanosheet host surface. The distribution of O element was also tested, and shown in Fig. S2. Fourier-transform infrared (FT-IR) spectra and X-ray photoelectron spectroscopy (XPS) were also conducted to analyze composite composition and structure, respectively (Figs. S3 and S4). All these results showed that Ag and Br were presented in the form of Ag/AgBr. Thus, Ag/AgBr NPs were dispersed on g-C_3_N_4_ surface uniformly and tend to combine with g-C_3_N_4_ thin nanosheets firmly and then formed heterojunction systems. Meanwhile, high-resolution XPS spectrum of O elemental was also tested, and shown in Fig. S5.

### Phase structure analyses

**Figure 4:**
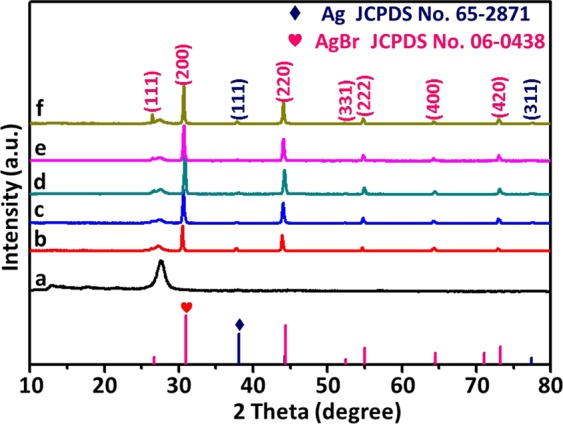
XRD patterns of (**a**) g-C_3_N_4_, (**b**) 5%Ag/AgBr/g-C_3_N_4_, (**c**) 10%Ag/AgBr/g-C_3_N_4_, (**d**) 15%Ag/AgBr/g-C_3_N_4_, (**e**) 18%Ag/AgBr/g-C_3_N_4_, and (**f**) 21%Ag/AgBr/g-C_3_N_4_; and the standard diffraction patterns of Ag (JCDPS No. 65–2871) and AgBr (JCDPS No. 06–0438).

The crystalline structures of all obtained photocatalysts were characterized by X-ray diffraction (XRD) and shown in Fig. 4. Typical XRD pattern of g-C_3_N_4_ (Fig. 4a) showed an intense diffraction peak at 2θ = 27.7°, which could be indexed as (002) interlayer-stacking peak corresponding to an interlayer distance of d = 0.32 nm, while the peak at 13.0° was (100) plane of hexagonal g-C_3_N_4_ (JCPDS card No. 87–1526), which represented an in-plane structural packing motif with a period of 0.675 nm^16^. As shown in Fig. 4b–f, the patterns corresponded to photocatalysts with an increasing Ag/AgBr mass ratio (5%, 10%, 15%, 18%, and 21%) were exhibited. After assembling Ag/AgBr NPs with g-C_3_N_4_ nanosheets, several new diffraction peaks (marked with “♥”) were observed at 26.5°, 30.7°, 44.1°, 52.3°, 54.8°, 64.3° and 73.0°, which were assigned to (111), (200), (220), (331), (222), (400) and (420) planes of AgBr crystal (JCPDS No. 06–0438)^33,39,46^. The diffraction peaks (marked with “◆”) of the metallic Ag at 37.8° and 77.4° were detected, which could be assigned to (111) and (311) crystal faces corresponding to the pattern of crystalline Ag, respectively (JCPDS No. 65–2871)^46^. The intensity of AgBr peaks were enhanced gradually with the increasing of AgBr amount, while the intensity of g-C_3_N_4_ peak decreased. The intensity of g-C_3_N_4_ peak decreasing regularly indicated that Ag/AgBr was compounded onto g-C_3_N_4_ surface. No other impurity phase was observed in the pattern of Ag/AgBr/g-C_3_N_4_. Thus, Ag/AgBr/g-C_3_N_4_ samples contained three phases, namely, Ag, AgBr and g-C_3_N_4_.

### Optical properties of the composites

**Figure 5:**
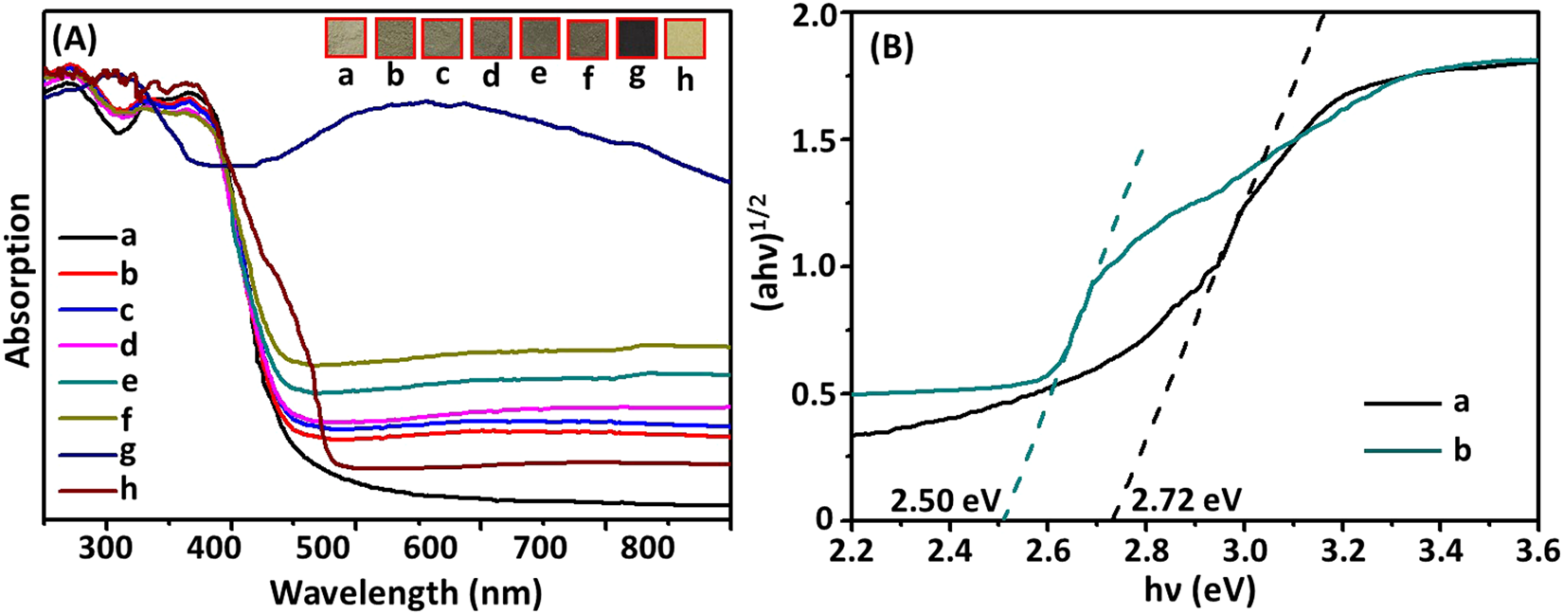
(**A**) UV-vis absorption spectra (in the diffuse reflectance spectra mode and the inset was the photo of samples) of (a) g-C_3_N_4_, (b) 5%Ag/AgBr/g-C_3_N_4_, (c) 10%Ag/AgBr/g-C_3_N_4_, (d) 15%Ag/AgBr/g-C_3_N_4_, (e) 18%Ag/AgBr/g-C_3_N_4_, (f) 21%Ag/AgBr/g-C_3_N_4_, (g) Ag/AgBr and (h) AgBr; and (**B**) Plots of (a) g-C_3_N_4_ and (b) 18%Ag/AgBr/g-C_3_N_4_ ((ahν)^1/2^ Vs. hν).

The optical properties of photocatalysts was determined by UV-vis diffuse reflectance spectrum (UV–vis DRS). As shown in Fig. 5A, pure g-C_3_N_4_ showed a typical semiconductor absorption in the range of 250–450 nm, which was rooted the charge-transfer from valence band (VB, occupied by N2p orbitals) to conduction band (CB, formed by C2p orbitals)^16,47^. The absorption thresholds of g-C_3_N_4_ was approximately 455 nm (shown as Fig. 5A(a)). Spectra of hybrid composites with different loading amount of Ag/AgBr (5%, 10%, 15%, 18% and 21%) were shown in Fig. 5A(b–f), the absorption thresholds of composites showed a negligible shift. The light absorption of Ag/AgBr/g-C_3_N_4_ was significantly increased in the wavelength range of 500–800 nm, and the absorption intensities increased according to the increase of Ag/AgBr content, which were attributed to SPR effect of Ag NPs. As shown in Fig. 5A(g), Ag/AgBr showed a strong absorption in all test region, thereby corresponding to the increasing by SPR effect of Ag NPs^48^. Meanwhile, the spectrum of AgBr was also tested and showed in Fig. 5A(h), the thresholds of AgBr was around 496 nm. It just showed a slight red shift to some extent compared to g-C_3_N_4_. We obtained that the enhancing absorption of composite photocatalysts in visible light region was mainly obtained from SPR effect of Ag. Meanwhile, band gap (E_g_) of g-C_3_N_4_ and AgBr could be calculated by Eq. (1) and obtained from UV-vis spectra, as follows^49^:

1 $$\alpha h\nu ={\rm{A}}{(h\nu -{E}_{g})}^{{\rm{n}}/2}$$

Where *α* is absorption coefficient, *hν* is photon energy, and A is proportionality constant^50^. Given the optical transition type of a semiconductor, *n* value was 1 (direct transition) or 4 (indirect transition). The *n* values of g-C_3_N_4_ and AgBr were both 4^51,52^. Hence, the plot of (*αhν*)^1/2^ versus photon energy (*hν*) for g-C_3_N_4_ and 18%Ag/AgBr/g-C_3_N_4_ was obtained, as shown in Fig. 5B. As a result, the band gap energies of AgBr and g-C_3_N_4_ were estimated from the plot of (*αhν*)^1/2^ versus energy (*hν*) curve. Thus, *E*_*g*_ values of AgBr and g-C_3_N_4_ were 2.50 and 2.72 eV (Fig. 5B), respectively.

### Visible light photocatalytic activities and evaluation of stability

**Figure 6:**
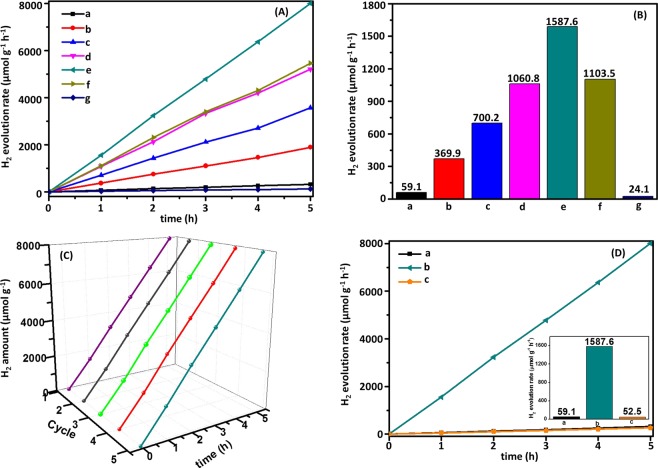
(**A**) Time courses of photocatalytic H_2_ (**B**) average rate of H_2_: (a) g-C_3_N_4_, (b) 5%Ag/AgBr/g-C_3_N_4_, (c) 10%Ag/AgBr/g-C_3_N_4_, (d) 15%Ag/AgBr/g-C_3_N_4_, (e) 18%Ag/AgBr/g-C_3_N_4_, (f) 21%Ag/AgBr/g-C_3_N_4_ and (g) Ag/AgBr under visible light irradiation; (**C**) Recyclability of 18%Ag/AgBr/g-C_3_N_4_ in 5-cycle experiments for H_2_ evolution under visible light irradiation; (**D**) Time courses of photocatalytic H_2_ (insert: the average rate of H_2_): (a) g-C_3_N_4_, (b) 18%Ag/AgBr/g-C_3_N_4_, (c) mixture of g-C_3_N_4_ and Ag/AgBr(18 wt%).

The amount of H_2_ production for all photocatalysts was detected with triethanolamine (TEOA) as sacrificial reagent under visible light irradiation illumination (λ > 420 nm). The peak areas of different samples at different test times were obtained with gas chromatograph (GC) test. According to the standard curve y = 165546x (as shown in Fig. S6; R^2^ = 0.9998, y: peak area value, x: H_2_ volume), the amount of H_2_ was calculated. Under the same condition, the control experiments were conducted. The test results indicated that insignificant amount of H_2_ was detected without photocatalysts, light irradiation or H_2_O. Meanwhile, the amount of H_2_ using Ag/AgBr as photocatalyst was also conducted under the same condition, and a slight amount of H_2_ was detected. The corresponding results for time-dependent (Fig. 6A) and average (Fig. 6B) photoinduced H_2_ evolution for different photocatalysts were shown (the values were listed in Table S1). H_2_ production using bare g-C_3_N_4_ as photocatalyst was 59.1 μmol g^−1^ h^−1^, which was significantly lower than that of heterogeneous catalysts Ag/AgBr/g-C_3_N_4_ with the highest efficiency of 1587.6 μmol g^−1^ h^−1^ that was attained using 18%Ag/AgBr/g-C_3_N_4_ as photocatalyst. This result was approximately 27-fold to that of pure g-C_3_N_4_. The low photocatalytic ability of pure g-C_3_N_4_ was due to its relatively poor visible light absorption performance and low photogenerated charge-separation efficiency. Photogenerated e^−^ of g-C_3_N_4_ was extremely difficult to transfer to active sites and unable to participate in H_2_ production^53,54^. Compared with other g-C_3_N_4_-based photocatalysts, H_2_ production rate of Ag/AgBr/g-C_3_N_4_ was significantly enhanced (the comparison results were shown in Table 1), which was ascribed to plasmonic effect of Ag NPs for enhancing absorption in visible light region and the high charge separation efficiency of heterostructures^54–57^. Pure Ag/AgBr with substantial agglomeration (with the size from 100 nm to >700 nm) showed a poor photocatalytic activity (24.1 μmol g^−1^ h^−1^). Nevertheless, bulk Ag/AgBr showed a high absorption performance in visible light region. Considering its heavy agglomeration, only few active sites were found, and photogenerated charges transferring to photocatalyst surfaces to reduce H^+^ became extremely difficult. Meanwhile, photogenerated charge separation efficiency decreased significantly because e^−^ and h^+^ needed to move long distance inside of bulk Ag/AgBr and additional charge recombination centers were also observed.

**Table 1:** Comparison of photocatalytic H_2_ production rate reported in the literatures with our work.

Sample	Efficiency (μmol g^−1^ h^−1^)	Co-catalyst	Light sourec	Reference
g-C_3_N_4_-600	3267	No	λ ≥ 395 nm	ref. ^4^
N,S-TiO_2_/g-C_3_N_4_	6340	No	λ ≥ 400 nm	ref. ^11^
g-C_3_N_4_	10.7	3% Pt	λ ≥ 420 nm	ref. ^16^
CNS-CN-2	45.5	No	λ > 420 nm	ref. ^17^
sulfur-doped mesoporous g-C_3_N_4_	1360	3% Pt	λ ≥ ≥ 420 nm	ref. ^19^
Al-TCCP-0.1Pt	129	No	Visible light	ref. ^21^
CdS/g-C_3_N_4_/CuS	1151.2	No	λ ≥ ≥ 420 nm	ref. ^24^
Graphene/C_3_N_4_	451	1.5% Pt	λ > 400 nm	ref. ^26^
g-C_3_N_4_	629	2%Hollow CoS_x_ Polyhedrons	λ > 400 nm	ref. ^27^
g-C_3_N_4_–Pt-TiO_2_	1780	No	λ ≥ 420 nm	ref. ^29^
AgI:Ag	2.7	No	λ > 400 nm	ref. ^34^
SrTiO_3_/g-C_3_N_4_	440	1% Pt	λ ≥ 420 nm	ref. ^54^
Graphitized polyacrylonitrile (g-PAN)	370	1.5% Pt	λ > 400 nm	ref. ^55^
CeO_2_/g-C_3_N_4_	73.12	0.5% Pt	λ ≥ 420 nm	ref. ^56^
g-C_3_N_4_-SrTiO_3_:Rh	2233	No	λ ≥ 415 nm	ref. ^57^
g-C_3_N_4_/Ag_2_CrO_4_	902.1	0.6% Pt	λ ≥ 420 nm	ref. ^78^
Ag/AgBr/g-C_3_N_4_	1587.6	No	λ ≥ 420 nm	Our work

Y. P. Che, *et al*. Table 1.

Ag/AgBr content also exhibited a considerable influence on H_2_ evolution efficiency. As Ag/AgBr content increased, H_2_ production rate increased first and then decreased. When Ag/AgBr content was above 21%, an excessive amount of Ag/AgBr distributing on the limit g-C_3_N_4_ surface began to agglomerate, and the size became greater than 200 nm. The tight binding and interaction between g-C_3_N_4_ and Ag/AgBr disappeared, and heterostructure was no longer observed. Ag and AgBr became the combination centers of photogenerated charges, and charge separation efficiency was significantly decreased.

The stability and reusability of 18%Ag/AgBr/g-C_3_N_4_ nanocomposite as a representative were evaluated with a recycling experiment for 5 h, and the corresponding results were shown in Fig. 6C (Table S2). The results indicated that 18%Ag/AgBr/g-C_3_N_4_ hetero-structure photocatalyst showed a high stability in photocatalytic H_2_ production. In whole 5-cycle experiment, the amount of H_2_ obtained increased steadily with an extension during the reaction time, and total H_2_ produced showed insignificant reduction. As a comparison, the mixture of g-C_3_N_4_ and Ag/AgBr with the same content (18 wt%) was tested to conduct H_2_ production under the same condition. As shown in Fig. 6D (the corresponding results were listed in Table S3), the mixture showed a similar photocatalytic activity with that of pure g-C_3_N_4_. The mixture did not show a high efficiency for H_2_ production when it was used as photocatalyst (52.5 μmol g^−1^ h^−1^). This mixture was only obtained via a simply mechanical mixing of g-C_3_N_4_ and Ag/AgBr, they could not act synergistically. Photogenerated charges separation efficiency could not be increased to enhance photocatalyst activity. Meanwhile, the stability of 18%Ag/AgBr/g-C_3_N_4_ catalyst was further verified through XRD and SEM after 5 cycles of photocatalytic experiments (Figs. S7 and S8). The XRD result showed that the positions and intensity of peaks remained consistent, thereby indicating that the crystal structure of photocatalyst did not change significantly after photocatalytic experiments. SEM result revealed that morphology was steady. After 5-cycle photocatalytic test, Ag/AgBr NPs were still anchored firmly on g-C_3_N_4_ surface, thereby suggesting that the hetero-composite had high stability for photohydrogen production. Such high stability resulted from the formation of heterostructure between g-C_3_N_4_ and Ag/AgBr.

### Charge transfer properties

**Figure 7:**
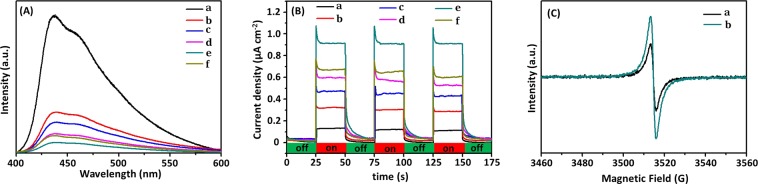
(**A**) Photoluminescence spectra: (a) g-C_3_N_4_, (b) 5%Ag/AgBr/g-C_3_N_4_, (c) 10%Ag/AgBr/g-C_3_N_4_, (d) 15%Ag/AgBr/g-C_3_N_4_, (e) 18%Ag/AgBr/g-C_3_N_4_, (f) 21%Ag/AgBr/g-C_3_N_4_; (**B**) Photocurrent responses: (a) g-C_3_N_4_, (b) 5%Ag/AgBr/g-C_3_N_4_, (c) 10%Ag/AgBr/g-C_3_N_4_, (d) 15%Ag/AgBr/g-C_3_N_4_, (e) 18%Ag/AgBr/g-C_3_N_4_, (f) 21%Ag/AgBr/g-C_3_N_4_; and (**C**) EPR spectra: (a) g-C_3_N_4_, (b) 18%Ag/AgBr/g-C_3_N_4_.

Photogenerated electron-hole separation efficiency of photocatalyst was an extremely important factor for photocatalytic activity, that was, high separation efficiency indicated high photocatalytic activity. Photoluminescence (PL) technique could effectively investigate migration, transfer, and recombination processes of photogenerated electron-hole pairs in semiconductors. PL spectra of photocatalysts should be investigated because only separated photo-induced e^−^ could be involved in subsequent photoreduction H_2_ evolution. PL emission spectra of photocatalysts were measured with an excitation light of *λ* = 315 nm, and the results were shown in Fig. 7A. Pure g-C_3_N_4_ had a wide and strong peak in PL spectrum excited at approximately 438 nm^17,19,58^. For Ag/AgBr/g-C_3_N_4_ hybrid materials, the position of emission peaks was similar to that of bare g-C_3_N_4_, but the intensity decreased significantly. This result indicated that Ag/AgBr/g-C_3_N_4_ composites had a considerably lower recombination rate of photogenerated charge carriers^19,59,60^. This result indicated that introduction of Ag/AgBr could significantly inhibit the recombination of photogenerated charges, which indicated that photogenerated e^−^ and h^+^ in Ag/AgBr/g-C_3_N_4_ heterostructure photocatalyst contained higher separation efficiency than those in bare g-C_3_N_4_. However, when Ag/AgBr content reached 21%, emission intensity of composites began to increase furtherly, thereby decreasing the photoactivity of photocatalyst. This phenomenon was due to the fact that excessive Ag/AgBr caused agglomeration of particles, thereby producing increased compound centers.

Photoelectrochemistry test could provide another powerful mean to study excitation and transportation of e^−^. To supply an evidence supporting the coupling of Ag/AgBr and g-C_3_N_4_ played an important role in photocatalytic H_2_ evolution, we tested photocurrent responses of g-C_3_N_4_ and Ag/AgBr/g-C_3_N_4_ with different contents and exhibited three on/off cycles of irradiation (Fig. 7B). Under Xe lamp irradiation, all tested working electrodes photocurrent responses were sharply increased once light source was turned on, and generated photocurrents were stable and reproducible during three on/off intermittent irradiation tests. Under the same test conditions, Ag/AgBr/g-C_3_N_4_ composites showed a higher photocurrent density than that of bare g-C_3_N_4_. The composite electrodes with 18% content of Ag/AgBr and pure g-C_3_N_4_ presented the highest (0.87 μA cm^−2^) and the lowest (0.13 μA cm^−2^) photocurrent density. Meanwhile, photocurrent responses of Ag/AgBr/g-C_3_N_4_ electrodes with other contents were between two extreme values mentioned above. The photocurrent density of Ag/AgBr/g-C_3_N_4_ composite electrodes increased gradually with the increase in Ag/AgBr content until the content achieved the optimized value and then began to decrease. The photocurrent results further confirmed that Ag/AgBr/g-C_3_N_4_ composites had higher separation efficiency of photogenerated electron-hole pairs than that of bare g-C_3_N_4_. This result indicated that Ag/AgBr had been effectively combined onto g-C_3_N_4_ surface, thereby leading to high separation efficiency of photogenerated electron-hole pairs in g-C_3_N_4_. The current density of composite electrode would not be further increased when its Ag/AgBr content reached the optimal value (18%). The enhancing effect to photocurrent density was impeded by the agglomeration of excess Ag/AgBr. Hence, separation efficiency of photogenerated charges and photocatalytic activity decreased. Ag NPs introduced by Ag/AgBr as a “trap” could capture a part of photogenerated electrons, showing in the results of photocurrent of doped catalyst electrodes there were a tailing phenomenon, thereby the introduction of Ag/AgBr into g-C_3_N_4_ further improved separation efficiency of photogenerated charge^61,62^. These results were consistent with the results of PL spectra.

Room-temperature electron paramagnetic resonance (EPR) spectra of g-C_3_N_4_ and Ag/AgBr/g-C_3_N_4_ were recorded (Fig. 7C). Both g-C_3_N_4_ and Ag/AgBr/g-C_3_N_4_ exhibit only one single lorentzian paramagnetic absorption signal with g value of 2.003, which was attributed to unpaired electrons in sp^2^-hybridized carbon atoms within the π-conjugated aromatic system^63,64^. However, Ag/AgBr/g-C_3_N_4_ showed an enhanced EPR signal as compared to g-C_3_N_4_, indicating the presence of more unpaired electrons in localized heterocyclic ring of hetero-catalyst due to the defects causing by coupling of Ag/AgBr^65,66^. That suggested more efficient generation of photoelectrons arising from composite photocatalyst^67^.

### Mechanism underlying enhanced photoactivity

**Figure 8:**
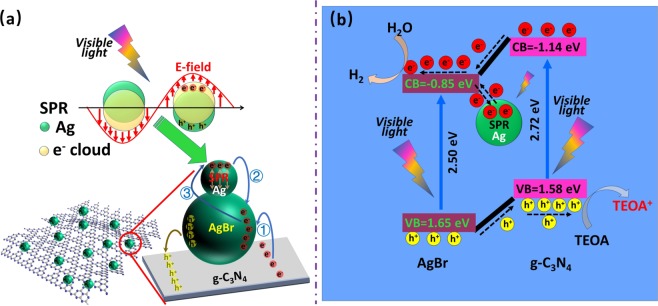
Schematic of the energy diagram and charge separation in ternary hybrid photocatalyst of Ag/AgBr/g-C_3_N_4_ photocatalysts under visible-light irradiation. (When the photocatalyst is excited by visible light irradiation, g-C_3_N_4_, AgBr and Ag were simultaneously excited to generate photogenerated electrons. Photogenerated electrons have three transmission paths: e^−^ on g-C_3_N_4_ CB could migrate to CB of AgBr; e^−^ generated by Ag nanoparticles with SPR effect also migrate to AgBr; meanwhile, a part of e^−^ from AgBr CB migrated back to Ag nanoparticles, which could ensure the stability of the catalyst. Finally, e^−^ were enriched on the surface of AgBr particles and participated in the reduction reaction to produce H_2_).

On the basis of results and analysis above, the mechanisms for photoreduction hydrogen evolution from water splitting in this system was proposed. The effects of enhancement by introducing Ag/AgBr into g-C_3_N_4_ forming a hetero-structure plasmonic photocatalyst were illustrated in Fig. 8. The band edge potential position played an important role in studying the flowchart of photoexcited charges. The potentials of CB and VB edges could be evaluated by Mulliken electronegativity theory, as follows^68^:

2 $${E}_{CB}=X-{E}_{C}-0.5{E}_{g}$$

3 $${E}_{VB}={E}_{CB}+{E}_{g}$$

where *X* is absolute electronegativity of the atom semiconductor (the geometric mean of absolute electronegativity of constituent atoms: the arithmetic mean of atomic electro affinity and the first ionization energy), and the *X* values for g-C_3_N_4_ and AgBr were 4.72 eV and 4.90 eV, respectively^32,69^; *E*_*c*_ is the energy of free e^−^ with the hydrogen scale (*E*_*c*_ = 4.5 eV); *Eg* is the band gap of semiconductor, and the band gap values of g-C_3_N_4_ and AgBr were 2.72 and 2.50 eV, respectively. As a result, the CB and VB values of g-C_3_N_4_ were -1.14 eV, 1.58 eV; the corresponding values of AgBr were -0.85 eV, 1.65 eV respectively.

As we know, SPR effect of noble metal nanocomposites produces a strong local electrostatic electric field, which could effectively suppress electron-hole recombination and improve efficiency of electron-hole separation^40,69^. In this system, the enhancement of composite photocatalytic performance was mainly attributed to its high separation efficiency due to easy transfer of e^−^ and h^+^ on heterojunction interfaces and the enhancement of light absorption caused by plasmon effect of Ag NPs^70^. When AgBr NPs were combined with g-C_3_N_4_, these two different semiconductor materials were connected closely, and heterojunction structure was obtained, which resulted in an effective photogenerated charge separation between these two semiconductors. Meanwhile, Ag NPs with a strong absorption around 450 nm adhering on AgBr surface could give out e^−^ by absorbing visible light due to its SPR effect, thereby further increasing photocatalytic activity by enhancing light absorption^71^. In this photocatalyst system, both g-C_3_N_4_ and AgBr could be excited simultaneously to generate e^−^ and h^+^ under visible light irradiation. Simultaneously, Ag NPs could absorb visible light and generate e^−^ due to its SPR effect^20,39,51,59^. Photogenerated e^−^ in g-C_3_N_4_ CB could easily transfer to AgBr CB, and h^+^ in AgBr VB could transfer to g-C_3_N_4_VB due to their matched energy levels. Electrons could transfer from Ag to AgBr because n-type AgBr semiconductor had a higher work function (AgBr = 5.3 eV, Ag = 4.25 eV) and a lower Fermi level than that of Ag^39^. e^−^ transferred from photoexcited metal Ag to CB of AgBr, thereby leaving positively charged Ag NPs^n+^ ^39,72,73^. The separated e^−^ could react with H_2_O to generate H_2_. At the same time, parts of e^−^ tended to be involved in the positive potential Ag NPs^n+^ and combined with Ag NPs^n+^ to form Ag NPs and sustained the stability of photocatalyst^74,75^. Finally, generated h^+^ could react with TEOA and OH^−^ on VB of g-C_3_N_4_.

In conclusion, polymeric compound g-C_3_N_4_ composed of graphitic planes that were constructed from tri-s-triazine units; when Ag/AgBr particles were loaded on g-C_3_N_4_ surface, Ag/AgBr/g-C_3_N_4_ interface was formed, which could effectively suppress photo-generated charges recombination, and e^−^ generated from g-C_3_N_4_ could transfer to Ag/AgBr nanoparticles efficiently. The synergistic effects of Ag/AgBr and g-C_3_N_4_ coupling enhanced electron transfer between the interfaces of Ag/AgBr and g-C_3_N_4_, thereby enhancing photocatalytic performance of Ag/AgBr/g-C_3_N_4_ photocatalysts. g-C_3_N_4_ could also act as a substrate, which was helpful in assembling Ag/AgBr on g-C_3_N_4_ surface to form hetero-structure. This nanostructure was especially favorable for photocatalytic applications because highly dispersion nanoparticles and thin nanosheet could decrease the recombination amount of photo-generated charges in inner of Ag/AgBr and g-C_3_N_4_. Meanwhile, thin nanosheet of g-C_3_N_4_ allowed easy transfer of photogenerated e^−^ to g-C_3_N_4_ surface. Ag/AgBr that was formed on g-C_3_N_4_ surface *in situ*, significantly enhanced the visible light absorption of hybrid materials, thereby indicating that it could enhance visible-light energy utilization.

## Conclusions

We synthetized a ternary visible-light-response plasmonic photocatalyst Ag/AgBr/g-C_3_N_4_ by means of green and handy route. Prepared metal-semiconductor ternary composite photocatalyst exhibited a high visible-light photocatalytic activity for water splitting to generation hydrogen without Pt co-catalyst. According to experiment results, 18%Ag/AgBr/g-C_3_N_4_ showed the highest photoactivity, 1587.6 μmol g^−1^ h^−1^. Such excellent performance should be attributed to the distinctive heterojunction system, which could successfully transfer electron generated from g-C_3_N_4_ resulting in the suppression of electron-hole recombination. The coupling of these materials solved the problems of high electron-hole recombination rate of g-C_3_N_4_ and the large particle size of Ag/AgBr at the same time. Meanwhile, light absorption was also enhanced due to SPR effect of Ag NPs. And what’s more, this study opens up new insights into the design and preparation of novel ternary hybrid photocatalysts with high photoactivity and further utilizations in the field of energy and environment.

## Experimental

### Preparation

Urea (chemical grade) was purchased from Sinopharm Chemical Reagent Beijing Co., Ltd. 1-Allyl-3-methylimidazolium bromide ([Amim]Br, 97%), silver nitrate (AgNO_3_, 99%) and triethanolamine (TEOA, 98%) were obtained from Aladdin Industry Corporation. All of these chemicals were used as they were gotten. Deionized water was obtained from pure water system (GWA-UN, Beijing, China).

Pure g-C_3_N_4_ was prepared via a simple calcined method, with urea as precursor^76^. First, 10 g of urea was placed into an alumina crucible with a lid, heated to 600 °C at a rate of 5 °C min^−1^, and maintained at this temperature for 4 h in a static air atmosphere. Then, the product was collected when cooled down to room temperature slowly. Light-yellow sample was dried at 50 °C after washing with water and ethanol. The obtained g-C_3_N_4_ was grinded for further experiments.

Ag/AgBr/g-C_3_N_4_ composites were synthetized by an improved deposition-precipitation, followed *in situ* photoreduction^77^. Typically, 85.6 mg as-prepared g-C_3_N_4_ powders were immersed into 40 mL of deionized water and sonicated for 60 min. Then, AgNO_3_ solution (17 mg, 20 mL) was dropped under dark condition, and amino groups on g-C_3_N_4_ sheet surface could coordinate with Ag ions tightly. Afterward, [Amim]Br solution (20.3 mg, 20 mL) was added into the g-C_3_N_4_ suspension by dropping under vigorous stirring and reacting for 4 h under dark condition. Considering the steric hindrance of [Amim]^+^, AgBr was obtained slowly by the action between Br^−^ and Ag^+^. Then, the reaction system was irradiated by a 500 W high-pressure Hg lamp (*λ* = 365 nm) for 15 min under stirring to obtain a spot of Ag NPs. The precipitate was collected by centrifugation, washed with ethanol and water several times, and vacuum dried at 60 °C for 12 h. Finally, Ag/AgBr/g-C_3_N_4_ composite with a theoretical mass ratio of Ag/AgBr to g-C_3_N_4_ at 18:82 was obtained and nominated as 18%Ag/AgBr/g-C_3_N_4_. Ag/AgBr/g-C_3_N_4_ composites with other Ag/AgBr content were prepared by changing the amount of [Amim]Br and AgNO_3_, and named as 5%Ag/AgBr/g-C_3_N_4_, 10%Ag/AgBr/g-C_3_N_4_, 15%Ag/AgBr/g-C_3_N_4_, 21%Ag/AgBr/g-C_3_N_4_. Ag/AgBr was prepared by a similar method without g-C_3_N_4_. AgBr was synthesized without g-C_3_N_4_ and UV light irradiation.

### Characterization

The morphologies were determined by field-emission scanning electron microscope (FESEM, JSM-7500F, Electron Optics Laboratory Co., Ltd., Japan) and transmission electron microscopy (TEM, Tecnai G^2^ 20 S-TWIN, FEI, USA). Energy dispersive spectroscopy (EDS) data were collected by FESEM equipped with EDS accessories (INCA Energy 250, Oxford, USA). The crystalline structure of samples was analyzed by X-ray diffraction (XRD, XRD-6000, Shimadzu, Japan). The survey and high-resolution spectra data of photocatalysts were obtained from X-ray photoelectron spectroscopy (XPS, ESCALAB 250Xi, Thermo Fisher, USA). The absorption spectra under a UV-vis diffuse reflectance spectrum (UV-vis DRS) mode were recorded on UV-vis spectrophotometer (UV-3600, Shimadzu, Japan) in a range of 250–800 nm. Fourier-transform infrared (FT-IR) spectra were conducted on Nicolet iS10 IR spectrophotometer (Thermo Scientific, USA). The photoluminescence (PL) data were recorded by Hitachi F-4500 fluorescence spectrometer (Japan, photomultiplier tube voltage of 400 V) with a scanning speed of 240 nm min^−1^. The electron paramagnetic resonance (EPR) spectra were carried out by A300-10/12 (Bruker, Germany).

### Photocatalytic activity of H_2_ evolution

The activity of as-prepared photocatalysts was verified through photocatalytic H_2_-evolution experiments. First, 50 mg photocatalyst dispersing in 100 mL of solvent (90 mL of deionized water and 10 mL of TEOA) was sealed into a top-irradiation quartz vessel. Second, the reaction vessel was installed to gas-closed circulation system (Labsolar-6A, Beijing Perfectlight Technology Co., Ltd., China) and vacuum pumped to avoid the adverse effects of dissolved air in the system. Third, a 300 W Xe lamp (light intensity: 100 mW cm^−2^; Microsolar300, Beijing Perfectlight Technology Co., Ltd., China) with a 420 nm cut-off filter was turned on to illuminate the system under stirring. The amount of gas was detected *in situ* through an online gas chromatograph (type: GC7900, Tech-comp Shanghai Co., Ltd., China) with Ar as carried gas to determine the amount of produced H_2_.

### Photoelectrochemical measurement

The working electrode was prepared according to our previous method^78^. Briefly, 230 μL of water, 250 μL of ethanol, and 20 μL of Nafion (5 wt%) were mixed and stirred for 20 min. Second, 10 mg as-prepared photocatalyst was placed into the solution above, dispersed ultrasonically for 30 min, and then stirred overnight. Afterward, 20 μL of the resulting colloidal dispersion was dispersed onto the surface of clear ITO (with a size of 1 × 1 cm). Lastly, the electrodes were placed into a culture vessel and dried under an ambient temperature for 4 h. The transient photocurrent was obtained from a standard three-electrode system on an electrochemical workstation (CHI 660D, Shanghai Chen Hua Instrument Co., Ltd., China); Pt plate was used as counter electrode, and Ag/AgCl electrode (in saturated KCl solution) served as a reference electrode. A solar simulator illumination (light intensity: 100 mW cm^−2^, CXE-350, Photoelectric Instrument Factory of Beijing Normal University, China) was used as the light source.

## Supplementary information

Supplementary information 
