# Supplementary material for: Plasmonic ternary hybrid photocatalyst based on polymeric g-C3N4 towards visible light hydrogen generation
Source: Sci Rep. 2020 Jan 20;10:721. doi: 10.1038/s41598-020-57493-x (PMC6971283; doi:10.1038/s41598-020-57493-x)
Supplement: Supplementary file 1 — Supplementary information [file 41598_2020_57493_MOESM1_ESM.pdf]

Plasmonic ternary hybrid photocatalyst based on polymeric g-C_3N_4 towards visible light hydrogen generation

Yuping Che[1], Yang Wang[3], Bingxin Lu[1], Jin Zhai[1*], Kefeng Wang[2*], and Zhaoyue Liu[1]

[1]Key Laboratory of Bio-Inspired Smart Interfacial Science, Technology of Ministry of Education and Beijing Advanced Innovation Center for Biomedical Engineering, Beijing Key Laboratory of Bio-inspired Energy Materials and Devices, School of Chemistry, Beihang University, Beijing 100191, P. R. China

[2]Henan Engineering Center of New Energy Battery Materials, College of Chemistry and Chemical Engineering, Shangqiu Normal University, Shangqiu 476000, Henan, P. R. China

[3]Institute of Chemistry Chinese Academy of Sciences, Beijing 100190, China

*Corresponding Author: zhaijin@buaa.edu.cn; wangkf2007@163.com

1. Scanning Electron Microscope (SEM) Analysis

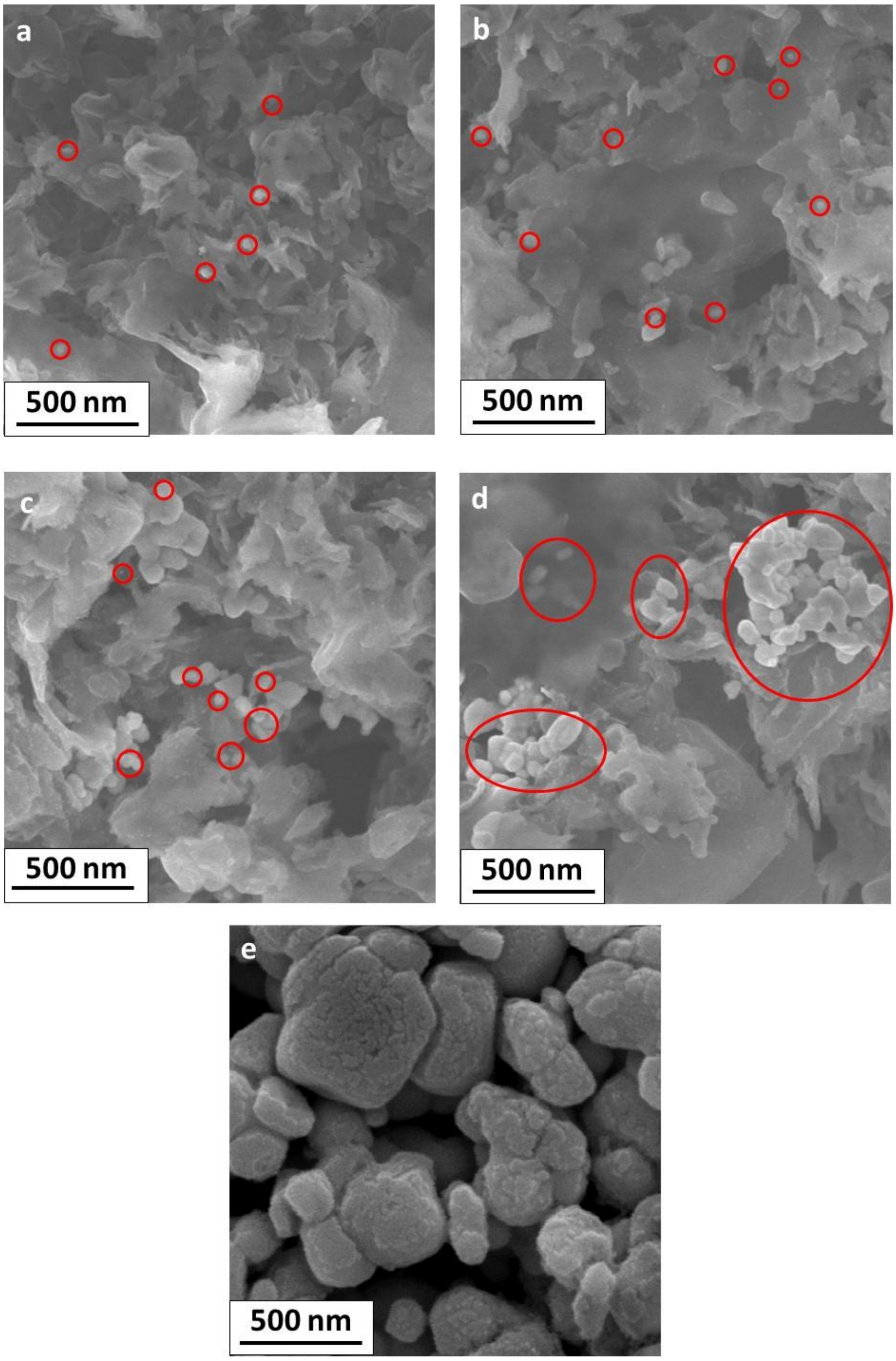

Figure S1 SEM images of (a) 5%Ag/AgBr/g-C_3N_4, (b) 10%Ag/AgBr/g-C_3N_4, (c) 15%Ag/AgBr/g-C_3N_4, (d) 21%Ag/AgBr/g-C_3N_4, and (e) Ag/AgBr

2. Energy Dispersive Spectroscopy (EDS) Analysis

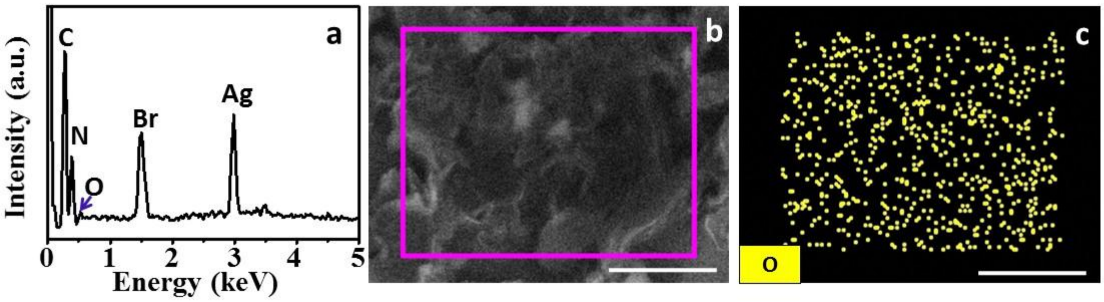

Figure S2 (a) EDS spectra for the 18%Ag/AgBr/g-C_3N_4, (b) SEM of 18%Ag/AgBr/g-C_3N_4, (c) EDS mapping for O element (the scale bar is 500 nm)

3. Fourier-transform infrared (FT-IR) spectra

Figure S3 depicted the FT-IR spectra of the bare g-C_3N_4 and Ag/AgBr/g-C_3N_4 composites with different contents. In the case of pure g-C_3N_4, the strong bands which corresponding to the feature-distinctive stretch modes of aromatic CN heterocycles in the 1200-1650 cm^{-1} region were found in the spectrum. The absorption bands near at 1562 and 1639 cm^{-1} were attributed to C=N stretching, while the other four bands at 1240, 1319, 1410 and 1456 cm^{-1} were attributed to aromatic C-N stretching. Additionally, the characteristic breathing mode of triazine units at 808 cm^{-1} was observed[8, 9]. A broad band near 3100 cm^{-1} corresponding to the stretching modes of terminal NH_2 or NH groups at the defect sites of the aromatic ring were also been observed. It could also be clearly seen that the main characteristic peaks of g-C_3N_4 appeared in all Ag/AgBr/g-C_3N_4 photocatalysts[8,10]. In the case of the Ag/AgBr/g-C_3N_4 composites, the characteristic peaks of g-C_3N_4 did not move after the introduction of Ag/AgBr nanoparticles. It was clear that the modification with Ag/AgBr did not alter the FT-IR spectrum of the g-C_3N_4, which indicated that there were no covalent bondz formed between Ag/AgBr and g-C_3N_4. The Ag/AgBr nanoparticles were deposited and well dispersed on the g-C_3N_4 surface, and the EDS, SEM and TEM characterization would be analyzed in detail.

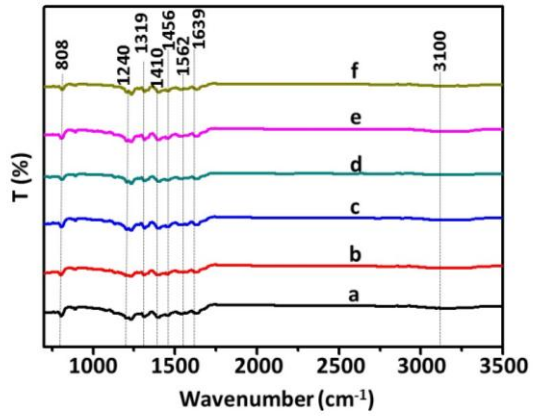

Figure S3 FT-IR spectra of (a) g-C_3N_4, (b) 5%Ag/AgBr/g-C_3N_4, (c) 10%Ag/AgBr/g-C_3N_4, (d) 15%Ag/AgBr/g-C_3N_4, (e) 18%Ag/AgBr/g-C_3N_4, and (f) 21%Ag/AgBr/g-C_3N_4

4. X-ray photoelectron spectroscopy (XPS) Analysis

In order to further ascertain in-depth, the information about the functional group and surface electronic state of pure g-C_3N_4 and 18%Ag/AgBr/g-C_3N_4 composite, the X-ray photoelectron spectroscopy (XPS) spectra were detected and shown in Figure S4. The survey spectrum in Figure S3Aa indicated that the main elements on the surface of the g-C_3N_4 sample were C, N and O. And it clearly showed that 18%Ag/AgBr/g-C_3N_4 composite (Figure S4Ab) consisted of C, N, Ag, Br and O elements. The appearance of O was due to the adsorption of O_2 onto the surface of the samples. The corresponding high-resolution XPS spectra were shown in Figure S4B-E. The XPS peaks of the C 1s were at 284.8 and 288.2 eV, as shown in Figure S3B, mainly derived from the g-C_3N_4 and the adventitious hydrocarbon of the XPS instrument. The peak at 398.6 eV for the pure g-C_3N_4 was identified as nitrogen atoms in C-N-C groups[1]. And the N 1s in the 18%Ag/AgBr/g-C_3N_4 had a little shift from 398.6 to 398.7 eV (Figure S4C), which indicated that the N chemical environment in the Ag/AgBr/g-C_3N_4 had changed. It also suggested the existence of the interaction between Ag/AgBr and g-C_3N_4, and the Ag/AgBr was combined with the N site in the g-C_3N_4. Figure S3D showed the high-resolution XPS spectra of Ag 3d spectrum, the two peaks located at 367.6 and 373.6 eV were attributed to Ag $3d_{5/2}$ and Ag $3d_{3/2}$, respectively, suggesting the presence of Ag^+ species for AgBr. The Ag $3d_{5/2}$ peak was further divided into two different peaks at 367.6 and 368.6 eV, and the Ag $3d_{3/2}$ peak was divided into two different peaks at 373.6 and 374.0 eV[2-4]. The peaks at 368.6 and 374.0 eV were attributed to metal Ag and the peaks at 367.6 and 373.6 eV were attributed to Ag^+ of AgBr. The spectrum of Br 3d in Figure S3E showed that the binding energies of Br $3d_{5/2}$ and Br $3d_{3/2}$ appeared at 68.3 and 69.1 eV, respectively[5-7]. The XPS results of Ag 3d and N 1s confirmed the existence of metal Ag (agree with the XRD result, as shown in Figure 4) and the interaction between Ag/AgBr and g-C_3N_4, respectively. In addition, the XPS spectrum of O 1s was presented in Figure S5.

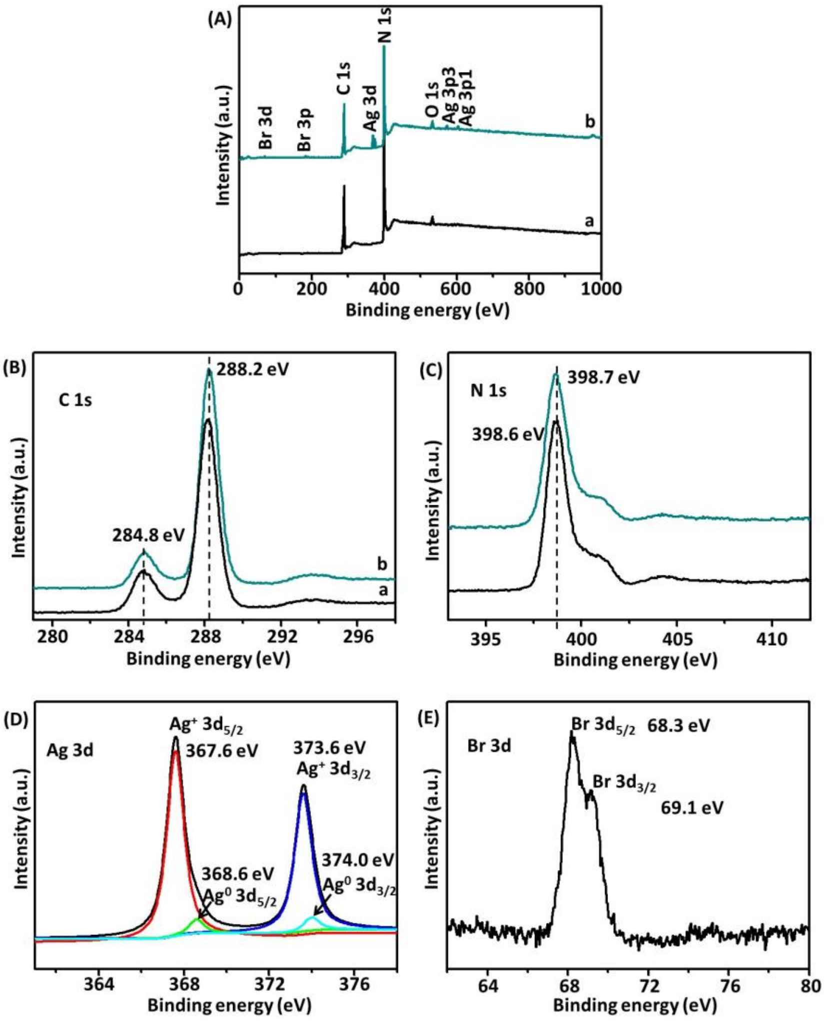

Figure S4 (A) XPS survey spectra of (a) g-C_3N_4, (b) 18%Ag/AgBr/g-C_3N_4. The corresponding high-resolution XPS spectra of the 18%Ag/AgBr/g-C_3N_4 composite: (B) C 1s, (C) N 1s, (D) Ag 3d, (E) Br 3d

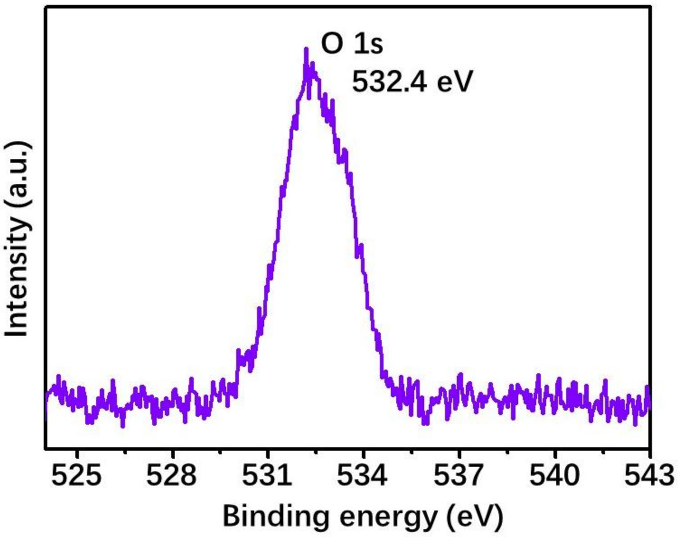

Figure S5 High-resolution XPS spectrum of 18%Ag/AgBr/g-C_3N_4 composite: O element

5. Analysis of Hydrogen Generation with Gas Chromatographic

The amount of hydrogen generation was determined by gas chromatography. Gas chromatographic conditions: the column type was 5A (15m*3 mm*3 mm, capillary column); the inlet temperature was 120 ℃; the column temperature was 60 ℃; the TCD temperature was 120 ℃； the carrier gas was Ar with a flow rate of 1 mL min^{-1}; the injection mode was splitless injection; the amount of injection was 1 mL; the current was 60 mA. We injected the same amount of standard gas containing different H_2 to get the following standard curve: y=165546x, R^2=0.9998 (as shown in Figure S6). We used the standard curve to calculate the hydrogen generation based on the measured peak area.

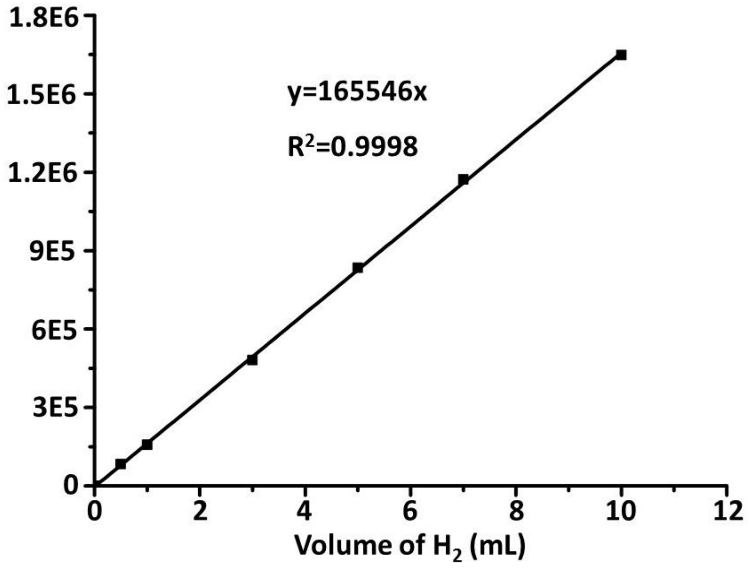

Figure S6 The standard curve of the amount of hydrogen generation

Table S1 The amount of hydrogen generation in 5 hours: (a) g-C_3N_4, (b) 5%Ag/AgBr/g-C_3N_4, (c) 10%Ag/AgBr/g-C_3N_4, (d) 15%Ag/AgBr/g-C_3N_4, (e) 18%Ag/AgBr/g-C_3N_4, and (f) 21%Ag/AgBr/g-C_3N_4.

sample	1h		2h		3h		4h		5h	
	Peak area (uV*s)	Amount of H_2 (mL/μmol)	Peak area (uV*s)	Amount of H_2 (mL/μmol)	Peak area (uV*s)	Amount of H_2 (mL/μmol)	Peak area (uV*s)	Amount of H_2 (mL/μmol)	Peak area (uV*s)	Amount of H_2 (mL/μmol)
a	233620	1.41/63	474658	2.87/128	697165	4.21/188	927065	5.60/250	1171783	7.08/316
b	1353520	8.18/365	2744088	16.58/730	4075343	24.62/1099	5399172	32.61/1456	7001153	42.29/1888
c	2599483	15.70/701	5276815	31.88/1423	7839185	47.35/2114	10012215	60.48/2700	13219859	79.86/3565
d	3978949	24.04/1073	7850329	47.42/2117	12340997	74.55/3328	15552304	93.95/4194	19282785	116.48/5200
e	5759947	34.79/1553	11985785	72.40/3232	17727216	107.08/4781	23602078	142.57/6365	29676967	179.27/8003
f	4086475	24.68/1102	8543780	51.61/2304	12593145	76.07/3396	15945375	96.32/4300	20213575	122.1/5451

Table S2 The stability of hydrogen generation for 18% $Ag/AgBr/g\text{-}C_3N_4$ in five cycles.

Cycle	1h		2h		3h		4h		5h	
	Peak area (uV*s)	Amount of H_2 (mL/μmol)	Peak area (uV*s)	Amount of H_2 (mL/μmol)	Peak area (uV*s)	Amount of H_2 (mL/μmol)	Peak area (uV*s)	Amount of H_2 (mL/μmol)	Peak area (uV*s)	Amount of H_2 (mL/μmol)
1st	5759947	34.79/1553	11985785	72.40/3232	17727216	107.08/4781	23602078	142.57/6365	29676967	179.27/8003
2nd	5592011	33.78/1508	11792172	71.23/3180	17680842	106.80/4768	23543555	142.22/6349	29554596	178.53/7970
3rd	5554929	33.56/1498	11784756	71.19/3178	17670002	106.74/4765	23569512	142.37/6356	29536055	178.42/7965
4th	5617969	33.94/1515	11870045	71.70/3201	17758715	107.27/4789	23573220	142.40/6357	29580553	178.68/7977
5th	5633818	34.03/1508	11787323	71.20/3179	17669818	106.74/4765	23548263	142.25/6350	29458199	177.95/7944

Table S3 The amount of hydrogen generation in 5 hours: mixture of g-C_3N_4 and Ag/AgBr (18%).

1h		2h		3h		4h		5h	
Peak area (uV*s)	Amount of H_2 (mL/μmol)	Peak area (uV*s)	Amount of H_2 (mL/μmol)	Peak area (uV*s)	Amount of H_2 (mL/μmol)	Peak area (uV*s)	Amount of H_2 (mL/μmol)	Peak area (uV*s)	Amount of H_2 (mL/μmol)
192814	1.16/51	396772	2.40/107	571057	3.45/154	782424	4.73/211	975282	5.89/263

6. Visible light photocatalytic stability

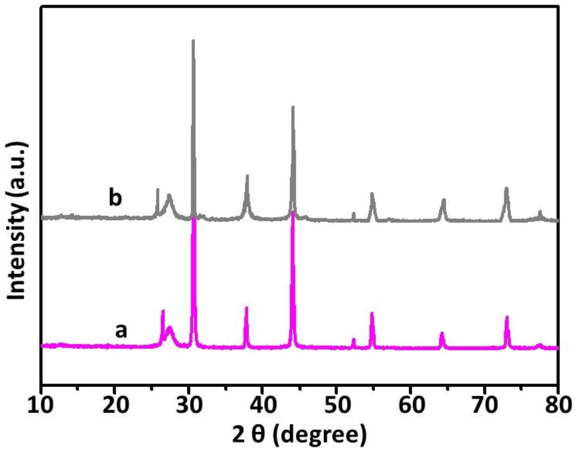

Figure S7 XRD patterns of 18%Ag/AgBr/g-C_3N_4 (a) fresh (b) used

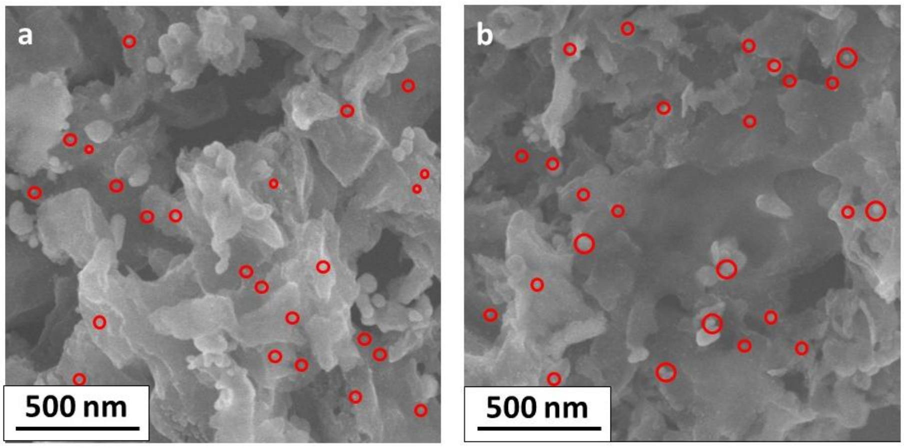

Figure S8 SEM of 18%Ag/AgBr/g-C_3N_4 (a) fresh (b) used

References

1. Han, Q., Wang, B., Zhao, Y., Hu, C. G. & Qu, L. T. A graphitic-C_3N_4 "seaweed" architecture for enhanced hydrogen evolution. *Angew. Chem. Int. Edit.* **54,** 11433-11437 (2015).

2. An, C. H., Peng, S. N. & Sun, Y. G. Facile synthesis of sunlight-driven AgCl: Ag plasmonic nanophotocatalyst. *Adv. Mater.* **22,** 2570-2574 (2010).

3. Hou, Y. *et al.* Ag_3PO_4 oxygen evolution photocatalyst employing synergistic action of Ag/AgBr nanoparticles and graphene sheets. *J. Phys. Chem. C* **116,** 20132-20139 (2012).

4. Dong, L. H. *et al.* Photoactivated route and new bromine source for AgBr/Ag nanocomposites with enhanced visible light photocatalytic activity. *Mater. Lett.* **91,** 245-248 (2013).

5. Cheng, H. F. et al. In situ ion exchange synthesis of the novel Ag/AgBr/BiOBr hybrid with highly efficient decontamination of pollutants. *Chem. Commun.* **47,** 7054-7056 (2011).

6. Kuai, L., Geng, B. Y., Chen, X. T., Zhao, Y. Y. & Luo, Y. C. Facile subsequently light-induced route to highly efficient and stable sunlight-driven Ag-AgBr plasmonic photocatalyst. *Langmuir* **26,** 18723-18727 (2010).

7. Wang, P. *et al.* Highly efficient visible light plasmonic photocatalyst Ag/Ag(Br, I). *Chem. Eur. J.* **16,** 10042-10047 (2010).

8. Kim, M., Hwang, S. & Yu J. S. Novel ordered nanoporous graphitic C_3N_4 as a support for Pt–Ru anode catalyst in direct methanol fuel cell. *J. Mater. Chem.* **17,** 1656-1659 (2007).

9. Li, X. Y. *et al.* Preparation and characterization of graphitic carbon nitride through pyrolysis of melamine. *Appl. Phys. A-Mater.* **94,** 387-392 (2008).

10. Yan, H. J., Yang, H. X. TiO_2-g-C_3N_4 composite materials for photocatalytic H_2 evolution under visible light irradiation. *J. Alloy. Compd.* **509,** L26-L29 (2011).
